# Flexibility of short ds-DNA intercalated by a dipyridophenazine ligand

**DOI:** 10.3389/fchem.2015.00025

**Published:** 2015-04-16

**Authors:** Fuchao Jia, Stéphane Despax, Jean-Pierre Münch, Pascal Hébraud

**Affiliations:** ^1^Institut de Physique et Chimie des Matériaux de Strasbourg/Centre National de la Recherche Scientifique, University of StrasbourgStrasbourg, France; ^2^Department of Physics, School of Science, Shangdong UniversityZibo, China

**Keywords:** DNA intercalation, DNA flexibility, FRET, ruthenium compounds, dppz intercalator

## Abstract

We use Förster Resonant Energy Transfer (FRET) in order to measure the increase of flexibility of short ds-DNA induced by the intercalation of dipyridophenazine (dppz) ligand in between DNA base pairs. By using a DNA double strand fluorescently labeled at its extremities, it is shown that the end-to-end length increase of DNA due to the intercalation of one dppz ligand is smaller than the DNA base pair interdistance. This may be explained either by a local bending of the DNA or by an increase of its flexibility. The persistence length of the formed DNA/ligand is evaluated. The described structure may have implications in the photophysical damages induced by the complexation of DNA by organometallic molecules.

## 1. Introduction

Since the first observation of the molecular “light switch” effect of *Ru*(*bpy*)_2_*dppz*^2+^ for DNA their interaction with DNA has been intensively studied (Friedman et al., 1990; Brennaman et al., 2002, 2004; Hu et al., 2009; Lim et al., 2009; Klajner et al., 2010; Sun et al., 2010; Song et al., 2012; Vidimar et al., 2012). *Ru*(*bpy*)_2_*dppz*^2+^ is a highly sensitive spectroscopic reporter of double-helical DNA (Very et al., 2012). In aqueous solution, luminescence is detectable when *Ru*(*bpy*)_2_*dppz*^2+^ intercalates into the nucleic acid structure (Batista and Martin, 2005). The emission properties (both in terms of intensity and spectral band) furthermore depend on the different helical forms of the polynucleotides, allowing the use of *Ru*(*bpy*)_2_*dppz*^2+^ as a sensitive, non-radioactive, luminescent DNA probe in both heterogeneous and homogeneous assays. *Ru*(*phen*)_2_*dppz*^2+^ possesses similar properties. Both complexes *Ru*(*bpy*)_2_*dppz*^2+^ and *Ru*(*phen*)_2_*dppz*^2+^ have served as “molecular light switch” for DNA, luminescing intensely in the presence of DNA but with no photoluminescence in aqueous solution and can be seen as unique reporters of nucleic acid structures (Song et al., 2012). The luminescent enhancement observed upon binding is attributed to the sensitivity of the excited state to quenching by water; the metal complex, upon intercalation into the DNA helix, is protected from the aqueous solvent, thereby preserving the luminescence (Friedman et al., 1990). Correlations between the extent of protection (depending upon the DNA conformation) and the luminescence parameters have been proven. Indeed, the strongest luminescent enhancement is observed for intercalation into DNA conformations which afford the greatest amount of overlap with access from the major groove, such as in triple helices. Differences are observed in the luminescent parameters between the two complexes which also correlate with the level of water protection. In the presence of nucleic acids, these two complexes exhibit biexponential decays in emission (Jenkins et al., 1992). Quenching studies are consistent with two intercalative binding modes for the dppz ligand in the major groove: one in which the metal-phenazine axis lies along the DNA dyad axis and another where the metal-phenazine axis lies almost perpendicular to the DNA dyad axis.

Upon binding to mismatched DNA base pair, *Ru*(*bpy*)_2_*dppz*^2+^ exhibits significant luminescent enhancements compared to well-matched DNA (Lim et al., 2009). In the presence of a single base mismatch, large luminescent enhancements are evident when ruthenium binds to an oligonucleotide containing an abasic site. Titrations with hairpin oligonucleotides containing a variable mismatch site have revealed correlation between the level of luminescent enhancement and the thermodynamic destabilization associated with the mismatch (Lim et al., 2009). This correlation is reminiscent of that found earlier for a bulky rhodium complex that binds mismatched DNA sites through metalloinsertion, where the complex binds the DNA from the minor groove side, ejecting the mismatched bases into the major groove (Lim et al., 2009). The smaller size of the dppz ligand also allows the ruthenium complex to bind through classical intercalation between two consecutive well-matched base pairs. Intercalated complexes are also located in the minor groove, stabilized by extensive ancillary interactions (Erkkila et al., 1999). This discrepancy notwithstanding, the crystal structure attests the remarkable structural flexibility of DNA upon high-density ligand binding, and illustrates the nuanced binding geometries sampled by a non-covalently bound small molecule. It highlights the dominance of metalloinsertion as the preferred binding mode to destabilized regions of DNA.

## 2. Flexibility

Although it is well-known that the length of DNA increases approximately linearly with the number of intercalated *Ru*(*bpy*)_2_*dppz*^2+^ molecules into the DNA double strand (Vladescu et al., 2007), the induced dynamical changes of the DNA chain have not been studied. Here, we quantify the variation of double strand flexibility induced by the intercalation of *Ru*(*bpy*)_2_*dppz*^2+^ into a short dsDNA (15 base pair long). We use Fluorescence Resonance Energy Transfer (FRET) in order to monitor the average distance between the extremities of a 15 bp dsDNA modified with two fluorophores at its extremities: both ends of dsDNA are modified by two types of fluorophores: Alexa488 and Alexa568. When Alexa488 is excited, it can decay by transferring non-radiative energy to Alexa568, which then de-excites by emitting photons of lower energy than those emitted by Alexa488. The efficiency of this energy transfer can be quantified from the measurement of the intensities emitted at low and high energy. It depends *a priori* on the coupling efficiency (and therefore the distance) between the two fluorophores. We will show that the increase of the average distance between the DNA extremities is incompatible with the assumption of a rigid and straight DNA/*Ru*(*bpy*)_2_*dppz*^2+^ complex. Nevertheless, the observation is made difficult due to the strong quenching of the fluorophores induced by the intercalation of the *Ru*(*bpy*)_2_*dppz*^2+^. Thus, in this article, from the analysis of the evolution of the lifetime of the donor on one hand, and that of the acceptor on the other hand, we deduce the evolution of the efficiency of the energy transfer in the concentration of ligand. We analyze the evolution of the emitted intensity of the donor and the lifetime of its excited state, and those of the acceptor, taking into account the photophysical properties of the ruthenium complex. We then obtain the evolution of the dsDNA average length as a function of the *Ru*(*bpy*)_2_*dppz*^2+^ complexation.

## 3. Materials and methods

**Figure 1:**
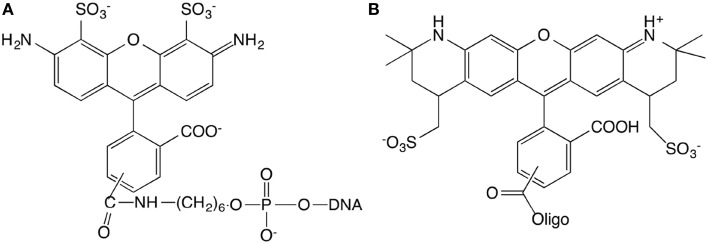
**Chemical structures of the Alexa488 (A) and Alexa568 (B) modified oligonucleotides**.

15 base pair dsDNA are used to perform the experiment. The number of DNA base pairs is limited by the range over which the FRET can take place that is approximately 10 nm. Complementary strands were purchased from IBA (Germany company) with sequences GGA GAC CAG AGG CCT and CCT CTG GTC TCC GGA. The length of 15 base pairs is 5.1 nm. The extremity of the short double-stranded DNA (dsDNA) is labeled with different kinds of fluorophores. The first sequence is modified in three different ways. (1) 5′-end is labeled with Alexa488, (2) 3′-end is labeled with Alexa568, (3) 5′-end and 3′-end are labeled with Alexa488 and Alexa568, respectively (Figure 1). The Alexa fluorophore is chemically linked with DNA base. Titrations are performed by the addition of *Ru*(*bpy*)_2_*dppz*^2+^ to a solution of DNA at constant DNA concentration, equal to 1μM.

A pulsed laser of 495 nm wavelength with a repetition rate of 1 MHz is used to excite the fluorophore. The photon detector temporal resolution is 0.1 ns. The wavelength collected is at 517 nm and 600 nm with 2 nm spectral slit. The acquisition time is chosen so that the number of photons collected in the first channel is equal to 10^4^. Under these experimental conditions, a measurement lasts between 10 and 120 min. All measurements are performed at constant room temperature (20°C).

The notations used in the analysis of the results are given in Table 1.

**Table 1:** **List of notations used in the analysis of the fluorophore emission**.

τ^*D*^	relaxation time of the donor in the absence of the acceptor
τ^*DA*^	relaxation time of the donor in the presence of the acceptor
τ^*A*^	relaxation time of the acceptor
τ^*A*^_600_	decay time of the emission of the acceptor at 600 nm
κ_*T*_, τ^*F*^	Förster Resonant Energy Transfer rate, τ^*F*^ = κ^−1^_*T*_
D*(*t*)	number of excited donor molecules at time *t*
D*_02_	initial number of excited donor molecules in the presence of the acceptor
D*_01_	initial number of excited donor molecules in the absence of the acceptor
A*(*t*)	number of excited acceptor molecules at time *t*
A*_02_	initial number of excited acceptor molecules in the presence of the donor
*N*^*D*^_517_	number of photons emitted at 517 nm by the donor in the absence of the acceptor
*N*^*DA*^_517_	number of photons emitted at 517 nm by the donor in the presence of the acceptor
*N*^*D*^_600_	number of photons emitted at 600 nm by the donor in the absence of the acceptor
*N*^*DA*^_600_	number of photons emitted at 600 nm by the donor in the presence of the acceptor
*N*^*AD*^_600_	number of photons emitted at 600 nm by the acceptor in the presence of the donor
*N*^*Ru*^_600_	number of photons emitted at 600 nm by the ruthenium compound

## 4. Analysis of the emission at 517 nm

We now turn to the analysis of the intensity emitted at 517 nm. The most important factor we care in FRET process is the transfer efficiency, that relies on two assumptions. First, it is assumed that the presence of the acceptor neither changes nor adds any relaxation process except for the FRET process. Then, the number donor molecules excited by the laser is assumed to remain constant when the acceptor is present. The transfer efficiency is defined as the probability that an excited donor fluorophore comes back to its ground state by FRET process. This can be expressed as:

(1) $$E=\kappa_{T}\tau^{DA}$$

where κ_*T*_ is the FRET transfer rate. κ_*T*_ is directly related to the distance *r* between the two fluorophores according to: $\kappa_{T}(r)=\frac{1}{\tau^{D}}{\left(\frac{R_{0}}{r}\right)}^{6}$, where *R*_0_ is the Förster distance and τ^*D*^ the relaxation time of the donor fluorophore in the absence of the acceptor. τ^*DA*^ is the overall characteristic decay time of the donor molecule in the presence of the acceptor: it is directly measured as the decay time of the fluorophore emission at its maximum wavelength. According to the first assumption, the presence of the acceptor does not change any relaxation process except for the FRET process. We thus have:

(2) $$\frac{1}{\tau^{DA}}=\frac{1}{\tau^{D}}+\kappa_{T}$$

where τ^*D*^ is the characteristic decay time in the absence of acceptor, that can also be measured directly. Then the transfer efficiency may be rewritten as:

(3) $$E=1-\frac{\tau^{DA}}{\tau^{D}}=\frac{1}{1+{\left(\frac{R_{0}}{r}\right)}^{6}}$$

D^*^ being the number concentration of excited donor molecules, we have:

(4) $$\frac{dD^{*}}{dt}=-\frac{D^{*}}{\tau^{DA}}$$

leading to:

(5) $$D^{*}(t)=D_{02}^{*}e^{-\frac{t}{1/\tau^{D}+\kappa_{T}}}=D_{02}^{*}e^{-t/\tau^{DA}}$$

where D^*^ is the number of excited donors at time *t*, D^*^_02_ is the initial population of excited donors in the presence of acceptor, τ^*DA*^ and τ^*D*^ are the lifetimes in the presence and absence of acceptor, respectively. Nevertheless, D^*^ cannot be measured directly: the emitted intensity is measured. But the donor emits photons both at 517 nm and at the maximum emission wavelength of this acceptor, at 600 nm (this is the cross-talk phenomenon).The number of photons emitted by the donor in the presence, *N*^*DA*^_517_, and in the absence,*N*^*D*^_517_, of the acceptor, at 517 can be expressed as the probability that their relaxation occurs through the emission of a photon at the corresponding wavelength:

(6) $$N_{517}^{D}\;=D_{01}^{*}\frac{\tau^{D}}{\tau_{517}^{D}}$$

(7) $$N_{517}^{DA}=D_{02}^{*}\frac{\tau^{DA}}{\tau_{517}^{D}}$$

where τ^*D*^ and τ^*DA*^ are the overall decay times in the absence and presence of the acceptor, τ^*D*^_517_ and τ^*D*^_600_ the characteristic decay times of photon emission at 517 nm and 600 nm by the donor. D^*^_01_ and D^*^_02_ are the initial number of excited donors by the laser in the absence and presence of acceptor. We thus have:

(8) $$\frac{N_{517}^{DA}}{N_{517}^{D}}=\frac{D_{02}^{*}}{D_{01}^{*}}\frac{\tau^{DA}}{\tau^{D}}=\frac{\tau^{DA}}{\tau^{D}}$$

assuming that the number of donor molecules excited by the laser does not change when the acceptor fluorophore is present. This leads to a new expression of the transfer efficiency:

(9) $$E=1-\frac{\tau^{DA}}{\tau^{D}}=1-\frac{N_{517}^{DA}}{N_{517}^{D}}$$

**Figure 2:**
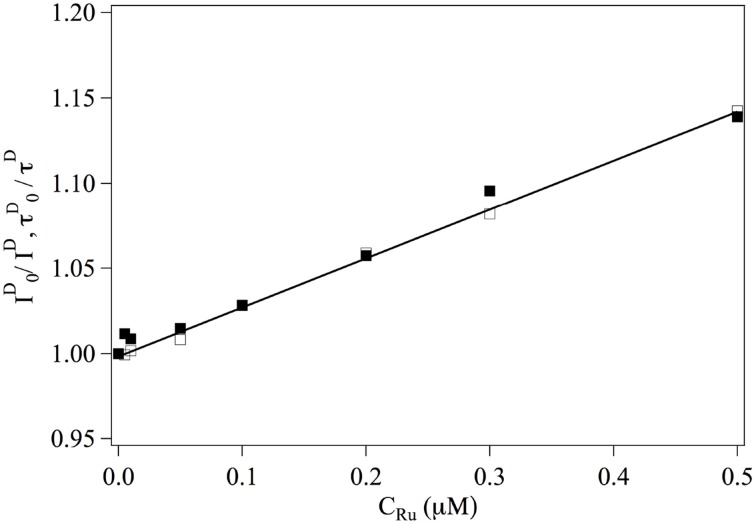
**Evolution of** $\frac{I_{0}}{I(C_{Ru})}$ **■ and** $\frac{\tau_{0}}{\tau (C_{Ru})}$ **□ as a function of the ruthenium concentration**. Continuous line is a adjustment fit of the data from which one gets *K*_*D*_ = 0.3 μM.^−1^.

We first consider the interaction between *Ru*(*bpy*)_2_*dppz*^2+^ and the fluorophores. The overall intensities and relaxation times of single labeled DNA molecules is observed to decrease slightly in the presence of *Ru*(*bpy*)_2_*dppz*^2+^, due to quenching. The inverse of the intensity emitted by the donor fluorophore as well as its life time are represented in Figure 2. One observes that:

(10) $$\frac{I_{0}}{I(C_{Ru})}=\frac{\tau_{0}}{\tau (C_{Ru})}=1+K_{D}C_{Ru}$$

where the subscript indicates that the measurement is performed at null *Ru*(*bpy*)_2_*dppz*^2+^ concentration. This relation implies that *Ru*(*bpy*)_2_*dppz*^2+^ dynamically quenches the emission of the donor fluorophore and defines the dynamical quenching constant. One finds *K*_*D*_ = 0.3 μM^−1^.

**Figure 3:**
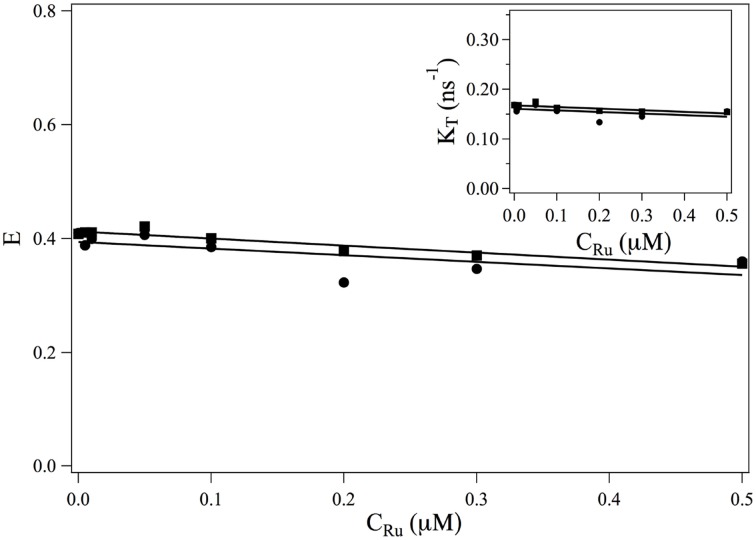
**Evolution of the FRET efficiency *E* and of κ_*T*_ = 1/τ_*F*_ (*insert*) as a function of the ruthenium concentration**. ■: values obtained from the measurements at 517 nm (Equations 9, 2). •: values obtained from the measurements at 600 nm (Equation 19) and κ_*T*_ = *E*/τ^*DA*^.

The transfer rate κ_*T*_ and the transfer efficiency *E* are separately computed from Equations 2, 9. The evolutions of $\tau^{F}=\frac{1}{\kappa_{T}}$ and *E* as a function of the ruthenium complex concentration are shown in Figure 3. On the whole, τ^*F*^ increases with the increase of the complex whereas the transfer efficiency decreases with increasing the *Ru*(*bpy*)_2_*dppz*^2+^ concentration. In the range of studied concentrations, the evolution of τ^*F*^ and *E* with the *Ru*(*bpy*)_2_*dppz*^2+^ concentration may be well-described by a linear behavior.

### 4.1. Dynamical measurements of the emission at 600 nm

The acceptor can be excited following two different mechanisms, either by direct excitation by the laser or by energy transfer from the donor fluorophore. τ^*A*^ is the overall relaxation time of the acceptor. The existence of FRET does not change the relaxation path of an excited acceptor, and τ^*A*^ is identical to the relaxation time of acceptor excited by the laser in the absence of donor. From the perspective of the acceptor, the FRET process can be determined by the following equations:

(11) $$\frac{dA^{*}}{dt}=\kappa_{T}D^{*}(t)-\frac{1}{\tau^{A}}A^{*}(t)$$

where the first term represents the excitation through FRET and the second one the desexcitation of the acceptor. With the initial condition A^*^(0) = A^*^_02_, the solution is:

(12) $$\begin{array}{l} A^{*}(t)=D_{02}^{*}\kappa_{T}\frac{1}{\tau^{A^{-1}}-\tau^{DA^{-1}}}e^{-t/\tau^{DA}}\\ \;+\left(A_{02}^{*}-D_{02}^{*}\kappa_{T}\frac{1}{\tau^{A}{}^{{}^{-1}}-\tau^{DA^{-1}}}\right)e^{-t/\tau^{A}} \end{array}$$

where A^*^_02_ is the number of acceptor molecules excited by the laser at time 0. This result states that the A^*^(*t*) should exhibit a two-time relaxation process, the first one equals to the relaxation time of the donor population in the presence of the acceptor, τ^*DA*^, and the second one equals to the relaxation time of the acceptor, τ^*A*^. Moreover, if $\frac{1}{\tau^{A}{}^{-1}-\tau^{DA}{}^{-1}}<0$, the amplitude of the acceptor decay over τ^*DA*^ is negative.

The number of photons emitted by the acceptor, *N*^*DA*^_600_ can be written as:

(13) $$\begin{array}{l} N_{600}^{AD}=\int_{0}^{\infty }\frac{1}{\tau_{600}^{A}}(D_{02}^{*}\kappa_{T}\frac{1}{\tau^{A}{}^{{}^{-1}}-\tau^{DA^{-1}}}e^{-t/\tau^{DA}}\\ \;+\frac{1}{\tau_{600}^{A}}(A_{02}^{*}-D_{02}^{*}\kappa_{T})\frac{1}{\tau^{A}{}^{{}^{-1}}-\tau^{DA^{-1}}}e^{-t/\tau^{A}}\text{​​})dt \end{array}$$

(14) $$=A_{02}^{*}\frac{\tau^{A}}{\tau_{600}^{A}}+D_{02}^{*}\kappa_{T}\tau^{DA}\frac{\tau^{A}}{\tau_{600}^{A}}$$

Where τ^*A*^_600_ is the characteristic time of photon emission at 600 nm by the acceptor, and τ^*A*^ = τ^*AD*^, since the presence of the donor does not change the relaxation of the acceptor. Finally, using *E* = κ_*T*_ τ^*DA*^, we have:

(15) $$N_{600}^{AD}=(A_{02}^{*}+D_{02}^{*}E)\frac{\tau^{A}}{\tau_{600}^{A}}$$

where A^*^_02_ is the number of excited acceptor from the lasers excitation, and D^*^_02_ *E* is the number of acceptor molecules excited by the FRET process.

We now consider the emission at 600 nm of double labeled dsDNA bound with ruthenium complex, *N*_600_. There are three contributions to this emission. (i) the contribution of the donor fluorophore, *N*^*DA*^_600_, the intensity emitted at 600 nm by the donor after a pulse, *I*^*DA*^_600_(*t*),(ii) the contribution of the acceptor, *N*^*AD*^_600_, the intensity emitted at 600 nm by the acceptor after a pulse, *I*^*AD*^_600_(*t*), and (iii) the emission of *Ru*(*bpy*)_2_*dppz*^2+^ that is luminescent when intercalated between DNA base pairs, *N*^*Ru*^_600_

So the total number of photons emitted at 600 nm can be expressed as *N*_600_ = *N*^*Ru*^_600_ + *N*^*DA*^_600_ + *N*^*AD*^_600_, whose we are going to perform an analysis term by term.

The intensity of emission at 600 nm of bound *Ru*(*bpy*)_2_*dppz*^2+^ has been measured when the ruthenium complex bound with non-labeled DNA. The contribution of the ruthenium complex is found to be smaller than 0.5% of the total observed intensity, so that we will neglect *N*^*Ru*^_600_ in the subsequent analysis.

The emission of the donor fluorophore at 600 nm in the presence of the acceptor cannot be measured independently and will be obtained indirectly. The number of photons emitted by the donor at the two studied wavelengths with and without the acceptor are given by the probabilities that the relaxation process occurs by the emission of a photon at the corresponding wavelength. We have (Equation 8):

(16) $$\frac{N_{517}^{DA}}{N_{517}^{D}}=\frac{D_{02}^{*}}{D_{01}^{*}}\frac{\tau^{DA}}{\tau^{D}}$$

and similarly at 600 nm:

(17) $$\frac{N_{600}^{DA}}{N_{600}^{D}}=\frac{D_{02}^{*}}{D_{01}^{*}}\frac{\tau^{DA}}{\tau^{D}}$$

We finally obtain the number of photons emitted by the donor in the presence of the acceptor, at 600 nm:

(18) $$N_{600}^{DA}=N_{517}^{DA}\frac{N_{600}^{D}}{N_{517}^{D}}$$

The ratio $\frac{N_{600}^{D}}{N_{517}^{D}}$ does not depend on the ruthenium complex concentration, and is measured at the null ruthenium concentration. It is equal to 2.74%. *N*^*DA*^_517_ is then measured directly from which *N*^*DA*^_600_ can be calculated as a function of the ruthenium concentration. We can then obtain the emission of the acceptor in the presence of the donor fluorophore, at 600 nm: $N_{600}^{AD}=N_{600}-N_{600}^{DA}=\left(A_{02}^{*}+D_{02}^{*}E\right)\frac{\tau^{A}}{\tau_{600}^{A}}$. The efficiency is then computed:

(19) $$E=\left(\frac{N_{600}^{AD}}{\tau^{A}}-\frac{A_{02}^{*}}{\tau_{600}^{A}}\right)\frac{\tau_{600}^{A}}{D_{02}^{*}}$$

We now determine the three ratios involved:

- $\frac{N_{600}^{AD}}{\tau^{A}}$: τ^*A*^ is the fluorescence lifetime of single labeled DNA with alexa568, and *N*^*AD*^_600_ has been obtained in the previous paragraph.
- $\frac{A_{02}^{*}}{\tau_{600}^{A}}$ is obtained from the amplitude of the 2 relaxation modes of the intensity emitted at 600 nm by two labeled DNA. This intensity is *I*_600_(*t*):
  (20) $$I_{600}(t)=I_{600}^{AD}(t)+I_{600}^{DA}(t)$$
  with
  (21) $$I_{600}^{DA}(t)=\frac{D_{02}^{*}}{\tau_{600}^{D}}e^{-t/\tau^{DA}}=\frac{N_{600}^{DA}}{\tau^{DA}}e^{-t/\tau^{DA}}$$
  and
  (22) $$I_{600}^{AD}(t)=\alpha_{1}e^{-t/\tau^{DA}}+\alpha_{2}e^{-t/\tau^{A}}$$
  which defines α_1_ and α_2_. They are obtained from the measurement of $I_{600}(t)=\left(\alpha_{1}+\frac{N_{600}^{DA}}{\tau^{DA}}\right)e^{-t/\tau^{DA}}+\alpha_{2}e^{-t/\tau^{A}}$. We thus obtain $\frac{A_{02}^{*}}{\tau_{600}^{A}}=\alpha_{1}+\alpha_{2}$.
- $\frac{\tau_{600}^{A}}{D_{02}^{*}}$ does not depend on the ruthenium concentration. Its value can not be measured from measurements of *I*_600_(*t*) as it always appears under the form $\frac{D_{02}^{*}}{\tau_{600}^{A}\kappa_{T}}$. The evolution of the efficiency E as a function of the ruthenium concentration is thus obtained up to a constant multiplicative factor. In order to compare the evolution of *E* (*C*_*Ru*_) with the efficiency obtained from measurement at 517 nm, we determine this multiplication factor at *C*_*Ru*_ = 0. The evolution of the FRET efficiency and of the transfer rate κ_*T*_ are plotted in Figure 3•.

## 5. Discussion

At the concentration under study, the fraction of DNA strand complexed by more than one ruthenium molecule may be neglected. We thus have a mixture of uncomplexed DNA and DNA strands complexed with one ruthenium molecule. Moreover this molecule may be intercalated at one or other of the 14 positions along the DNA double strand The intensities and lifetimes measured are thus average values of the intensities and lifetimes of these different complexes. The measured lifetime may be written as:

(23) $$\bar{\tau }=(1-\xi )\tau_{0}+\frac{\xi }{14}\sum_{n\;=\;1}^{14}\tau_{1,n}$$

where ξ is the fraction of DNA strands complexed with one *Ru*(*bpy*)_2_*dppz*^2+^, τ_0_ is the decay time of non-complexed DNA, τ_1, *n*_ is the decay time of DNA strand intercalated with *Ru*(*bpy*)_2_*dppz*^2+^ at the n^th^ position. We moreover assume that all the intercalation positions are equiprobable. The decay time may be expressed as the consequence of the existence of two processes:

(24) $$\frac{1}{\tau_{0}(C_{Ru})}=\frac{1}{\tau_{0}^{D}(C_{Ru})}+\frac{1}{\tau_{0}^{F}}$$

where τ^*D*^_0_ is the donor decay time in the absence of the acceptor at concentration *C*_*Ru*_, for a non-complexed dsDNA, and τ^*F*^_0_ = κ^−1^_*T*_ is the inverse of the FRET rate in the absence of complexation. τ^*F*^_0_ does not depend on the *Ru*(*bpy*)_2_*dppz*^2+^ concentration and is measured at *C*_*Ru*_ = 0.

(25) $$\frac{1}{\tau_{1,n}}=\frac{1}{\tau_{1,n}^{D}(C_{Ru})}+\frac{1}{\tau_{1,n}^{F}(C_{Ru})}$$

with similar notations. We have used the fact the τ^*D*^_1, *n*_(*C*_*Ru*_) does not depend on *n*, as the observed quenching is dynamic. We thus have:

(26) $$\bar{\tau }=(1-\xi )\frac{\tau_{0}^{F}\tau_{0}^{D}(C_{Ru})}{\tau_{0}^{F}+\tau_{0}^{D}}+\frac{\xi }{14}\tau_{1}^{D}(C_{Ru})\sum_{n\;=\;1}^{14}\frac{1}{1+{\left(\frac{R_{0}}{r_{1,n}}\right)}^{6}}$$

where *r*_1, *n*_ is the distance between the two fluorophores when the intercalation occurs at the *nth* position. It is known that the intercalation of the ruthenium compound induces a length increase of the DNA chain equal to the base pair distance. We moreover assume that the DNA double strand remains linear and rigid (Vladescu et al., 2007) Then, the length of the dsDNA, complexed with one *Ru*(*bpy*)_2_*dppz*^2+^ does not depend on the intercalation position and we have *r*_1, *n*_ = 16/15 *r*_0_ where *r*_0_ is the length of the 15 bp dsDNA chain. We now compute *r*_1_ from thee experimental measurements. We can use Equation 26, recognizing that:

(27) $${\left(\frac{r_{1}}{r_{0}}\right)}^{6}=\frac{\tau_{1}^{F}}{\tau_{0}^{F}}=\frac{\tau_{1}}{\tau_{0}^{F}\left(1-\frac{\tau_{1}}{\tau_{n}^{D}}\right)}$$

**Figure 4:**
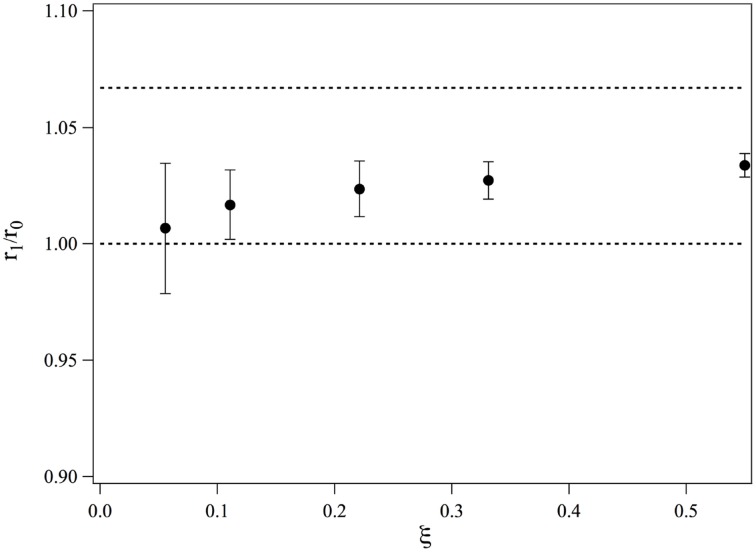
**Evolution of the average end-to-end distance of a 15-bp dsDNA bound to one molecule of *Ru*(*bpy*)_2_*dppz*^2+^, measured for different ratios of complexation**.

We obtain *r*_1_/*r*_0_. The values of *r*_1_/*r*_0_ are plotted in Figure 4 as a function of ξ. At low ξ, the bound ruthenium fraction is too low and the measurement is not accurate, but for values of ξ ≥ 0.2, *r*_1_/*r*_0_ saturates and its average value over the three highest complexation ratios is 1.028. This value is smaller than if the intercalation would have led to a length increase of a straight double strand, we would have: *r*_1, *n*_/*r*_0_ = 16/15 = 1.067. We thus conclude that the complexation induces a bending in the dsDNA. This bending may be static or dynamic, that is, due to an increase of flexibility of the DNA double strand at the intercalation. We define θ the bent angle induced by the intercalation, θ is of the order of 30°. It has been observed that short ds-DNA molecules are more flexible than worm-like chains of small lengths and whose persistence length would be equal to that of long DNA chains (Cloutier and Widom, 2005; Wiggins et al., 2006; Kahn, 2014; Le and Kim, 2014). This may also contribute to the increase of flexibility observed in our measurements.

As a conclusion, we have observed that the end-to-end distance increase of 15 bp dsDNA complexed with *Ru*(*bpy*)_2_*dppz*^2+^ is smaller than that would be increase if the DNA would remain rigid upon complexation. We may thus conclude that DNA bends upon complexation. Nevertheless, our experiments cannot determine whether time average bending is due to a local dynamic flexibility or a static kink induced by the intercalation.

### Conflict of interest statement

The authors declare that the research was conducted in the absence of any commercial or financial relationships that could be construed as a potential conflict of interest.
